# Monte Carlo simulation platform for laser Doppler flowmetry

**DOI:** 10.1117/1.JBO.30.8.087002

**Published:** 2025-08-26

**Authors:** David Thompson, Wietske Verveld, Guillaume Lajoinie, Michel Versluis, Wiendelt Steenbergen, Nienke Bosschaart

**Affiliations:** a University of Twente, TechMed Centre, Biomedical Photonic Imaging Group, Enschede, The Netherlands; b University of Twente, TechMed Centre, Physics of Fluids Group, Enschede, The Netherlands

**Keywords:** Monte Carlo simulations, light scattering, laser Doppler flowmetry, monodisperse suspensions, human milk

## Abstract

**Significance:**

Monte Carlo simulation of light propagation in turbid media is important in biomedical optics. Most existing platforms simulate light-tissue interactions in backscattering and planar geometries and are voxel-based, which limits their ability to model curved boundaries accurately. Few platforms incorporate Doppler shifts from flowing media, and they allow limited customization of flow profiles and scattering properties. Although laser Doppler flowmetry (LDF) is common in backscattering-based tissue measurements or low-scattering through-transmission setups, the intermediate case of through-transmission measurements in more scattering samples is underexplored. This case is relevant for applications such as flow quantification in lab-on-a-chip systems and inline flow sensors for biological fluids.

**Aim:**

To study flow in highly scattering samples (1 to $10\;{\mathrm{mm}}^{-1}$), we developed a voxel-free Monte Carlo simulation platform for through-transmission LDF: MC-Doppler. We compare simulated and experimental Doppler power spectra.

**Approach:**

MC-Doppler uses unit vectors and ray tracing to model light propagation, with fully customizable scattering phase functions and flow fields. It was tested with various suspensions of differently sized polystyrene beads, at flow rates ranging from 0 to 15  mL/min, within a 1 mm diameter glass tube.

**Results:**

Simulated and measured Doppler power spectra matched well for scattering coefficients up to $5\;{\mathrm{mm}}^{-1}$. Mismatches between the spectra were found near $10\;{\mathrm{mm}}^{-1}$.

**Conclusions:**

MC-Doppler accurately simulates light propagation for through-transmission laser Doppler up to moderate scattering coefficients.

## 1 Introduction

Since the mid-20th century,^1^^,^^2^ laser Doppler flowmetry (LDF) has been studied and applied to a range of applications in the mechanical and biomedical sciences.^3^^–^^5^ A major area of application for laser Doppler techniques is the noninvasive measurement of fluid flow. Since Stern’s seminal work in 1975,^6^ the measurement of blood perfusion in biological tissue has been a field of particular interest.

Two types of laser Doppler measurements can be distinguished in current literature: tissue perfusion measurements and *in vitro* flow measurements. In tissue perfusion measurements, a backscattering geometry is used. The flow direction in microvasculature is generally modeled as a random variable, with the assumption that most scattering events occur in the static tissue surrounding the microvessels.^7^^,^^8^ Multiple scattering in static tissue also randomizes the direction of incidence of the light onto any microvessels. It is often assumed that any Doppler-shifted light only experiences a single dynamic scattering event.^7^ Deeper penetration into tissue increases the chance of dynamic scattering.^9^^,^^10^

Most laser Doppler measurements in lab-based fluid flow systems have a well-defined flow direction, use low-scattering fluids, and are generally measured in through-transmission rather than backscattering.^11^^,^^12^ The incident light is also directional, being either a collimated or focused beam. These techniques generally rely on the interference pattern that is generated when a pair of beams overlaps in the focus of a lens or system of lenses. Furthermore, a reference beam is often used, either by use of the ballistic light transmitted through the sample tube or by bypassing the tube altogether.

Although the theory and experimental application of both these types of laser Doppler measurements are well known, the intermediate case of through-transmission measurements on relatively highly scattering samples in a flow tube has remained largely unexplored. Nevertheless, this specific case is of interest in a variety of biomedical applications, ranging from flow quantification in lab-on-a-chip and organ-on-a-chip systems^13^^,^^14^ to inline flow sensors for biological fluids. Our specific interest lies in the development of new methods for the inline characterization of human milk. Human milk has typical flow rates of 0.01 to 1  mL/s^15^^,^^16^ and scattering coefficients ($\mu_{s}$) from 2 to $30\;{\mathrm{mm}}^{-1}$.^17^^,^^18^ The light is mainly scattered by milk fat globules in the 1 to 10  μm size range.^19^ Nondestructive methods are preferred as human milk is a valuable resource and ideally should remain suited for infant consumption after analysis.^20^ A compact and unobtrusive tool to measure milk properties during pumping or feeding would provide access to valuable nutritional information with little to no energetic cost to mother and infant. Laser Doppler techniques are a prime candidate for such non-contact measurements on flowing scattering media such as milk.

For our situation, a highly scattering sample flowing in a tube, the usual method for through-transmission geometries is not suitable. A multitude of scattering events will distort both the focus and the interference pattern, meaning the signal is lost. Meanwhile, measuring in a backscattering configuration would mean a limited sampling depth as the concentration of scatterers increases. This would lead to an underestimation of the flow velocity as flow profiles are typically non-uniform. Although increased scattering can lead to loss of signal in a through-transmission geometry, this approach does sample the entire flow profile. To properly design and interpret any such experiment, Monte Carlo simulations are an important tool.

Many different Monte Carlo programs simulating light propagation in scattering media are available, for a range of different needs. MCML,^21^ for example, is designed to simulate multiple planar tissue layers to model light penetration through the skin into underlying tissues. More recent programs, such as MCXYZ,^22^ incorporate more general medium interface geometries and allow for the simulation of energy deposition from which thermal effects can also be simulated. Others focus on accessibility of the code for a broad audience of researchers and students by implementing more easily understandable, open-source programs such as MCmatlab.^23^ The programs mentioned so far do not have the in-built ability to simulate dynamic scattering, where Doppler shifts are induced by flowing media. Even without simulating the Doppler shifts, Monte Carlo simulations can still be useful to simulate the light propagation and thus yield information about the sampled volume. An example of this can be found in recent work by Zharkikh et al.,^24^ for a multimodal approach using LDF and fluorescence spectroscopy. Several programs suited to the simulation of Doppler shifts in tissue have also been developed. De Mul et al. did considerable work in using the simulations to characterize the behaviour of various configurations for LDF tissue measurements.^25^^–^^27^ Fredriksson’s work^28^^,^^29^ focused more on the development of quantitative metrics for the results of LDF measurements in tissue, based on the simulations.

Not all of the above-mentioned Monte Carlo programs are open source, or if they are, specialized programming skills are required to adapt them to incorporate Doppler shifts. Furthermore, in the programs that do allow for the simulation of Doppler shifts, the customisability of either the phase function, the flow profile and direction, or both are limited. The combination of scattering properties, as well as the flow profile and presence or absence of turbulence, can all influence the character of the Doppler power spectrum. Specialized tools are needed to quantify the volume flow in such a case, where one cannot rely on the inherent randomness of tissue-perfusion LDF to simplify the data processing. Finally, the vast majority of Monte Carlo programs for light transport in scattering media are voxel-based, leading to issues with nonplanar material interfaces, such as small flow tubes, due to the discretization. Although there are solutions to this problem,^30^ a non-voxel-based approach may be preferable in our case. The presence of very tight curvatures and shapes, which can be described by simple mathematics, means that forgoing voxelization can save processing time.

To this end, we have developed a new open-source MATLAB-based Monte Carlo platform named MC-Doppler, built to work with combinations of cylindrical and planar media, and with fully customizable flow fields and scattering properties. To deal with curved surfaces effectively and accurately, the platform is non-voxel-based, instead relying on unit vector operations and analytical solutions of intersections with medium interfaces. In this way, we can emulate many lab-based, medical, and industrial situations involving cylindrical cuvettes and flow tubes. As a first step toward a laser Doppler technique for flow characterization of highly scattering biological fluids, we demonstrate our Monte Carlo platform by simulating a series of suspensions of different monodisperse polystyrene particles under laminar flow conditions in a tube. The simulation results are compared with through-transmission LDF measurements.

## 2 Materials and Methods

### 2.1 Monte Carlo Simulations

**Fig. 1:**
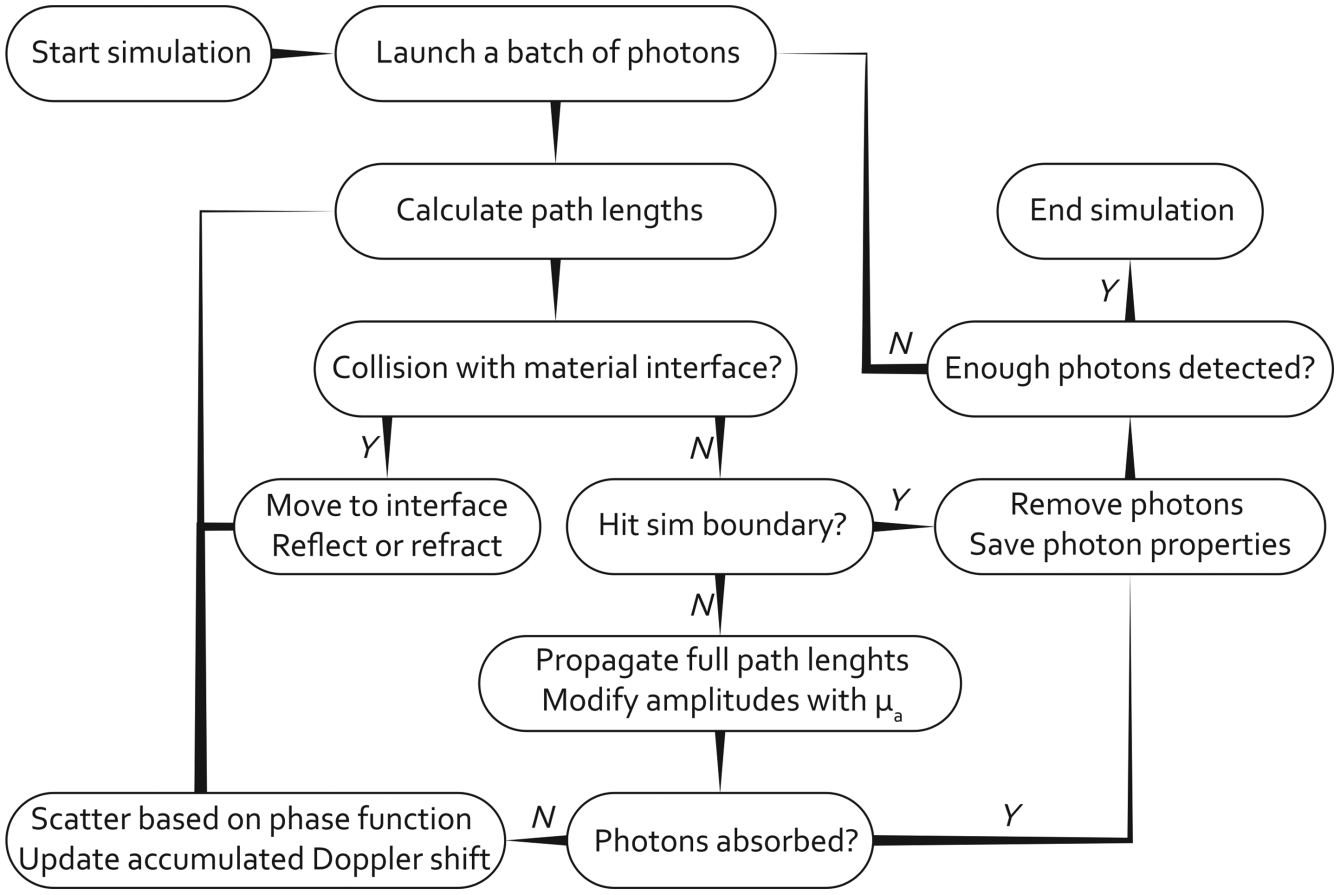
Flow chart of the main simulation loop of our Monte Carlo program.

MC-Doppler is open source and available on GitHub.^31^ It is written in MATLAB R2022b, which is well suited to using vector and matrix operations, and allows for easy GPU parallelization of processes. We do not rely on voxels, instead relying on vector operations to describe and modify the positions, directions, and polarization states of all photons. The flow chart in Fig. 1 shows a comprehensive overview of the operations performed by the program, the structure of which is inspired by Fredriksson’s work.^28^^,^^29^

#### 2.1.1 Setting the simulation parameters

To start a simulation, four structures need to be defined as an input into the program:

• The overall simulation properties: simulation boundary shape (spherical, cylindrical, or cubic) and dimensions, and the number of photons N to simulate. It also includes a Boolean, which determines whether or not to use the GPU.
• Media: the shapes (cylindrical, planar), dimensions, positions, optical properties (refractive index n, scattering coefficient $\mu_{s}$, and absorption coefficient $\mu_{a}$), and anonymous functions containing the flow fields of all media. Each property should be given as a 1×M array, where M is the number of media. Each subsequent entry in the media structure corresponds to a cylindrical medium of infinite length but finite diameter, counting from the outermost to the innermost medium. In this work, the cylindrical media are concentric, but they need not be and can be offset and tilted with respect to one another, though their boundaries should not overlap within the simulation area. The properties of any planar media are then appended to the end of the arrays. The planar media in the current version of MC-Doppler can have a finite thickness but must be infinite in extent in all other directions.
• Scattering angle selection functions per medium: The scattering inverse cumulative distribution functions (CDFs) are cubic smoothing splines derived from the scattering phase function. The phase function^32^ can be of any form, as long as it can be described as a 2×K matrix of values at scattering angles (θ) between 0 and π radians. The value of K should be sufficiently large to accurately interpolate in the phase function being used. Each of the two rows corresponds to the phase function for mutually orthogonal polarization states. For a polarization state between the two orthogonal states, the phase function is then given as a linear combination of the two, as in Eq. (1)^32^:
  $$P(\theta ,\phi )=\cos^{2}(\phi )P_{s}(\theta )+\sin^{2}(\phi )P_{p}(\theta ),$$ (1)
  where θ is in radians, ϕ is the azimuth, relative to the photon polarization direction, and $P_{s}$ and $P_{p}$ are the phase functions of the orthogonal polarization states. From this 2D matrix, a probability distribution function (PDF) for ϕ is found by summation over the dimension of θ. The PDF for ϕ is converted to an inverse CDF by numerically integrating, inverting, and generating a cubic smoothing spline with the MATLAB built-in *csaps* function. The same is done for θ for an appropriate number of values ($10^{2}$ to $10^{3}$) of ϕ between 0 and 2π, generating a matrix that can be interpolated for any value of ϕ between 0 and 2π and any value of θ between 0 and π when scattering angles are calculated. The advantage of using inverse CDFs is that they can be readily sampled by generating uniformly distributed random numbers between 0 and 1, for which MATLAB has an efficient built-in function. Inverse CDFs are not only used to calculate scattering angles but also to determine photon propagation path lengths elsewhere in the code.
• The light source properties: position, type [point source, pencil beam, Gaussian spot with finite numerical aperture (NA)], wavelength, directionality, spot size, and focus size depending on the precise type of source.

Once these structures have been defined, along with optional values such as the initial polarization state of the light, the simulation run can be initiated.

#### 2.1.2 Photon initialization

**Fig. 2:**
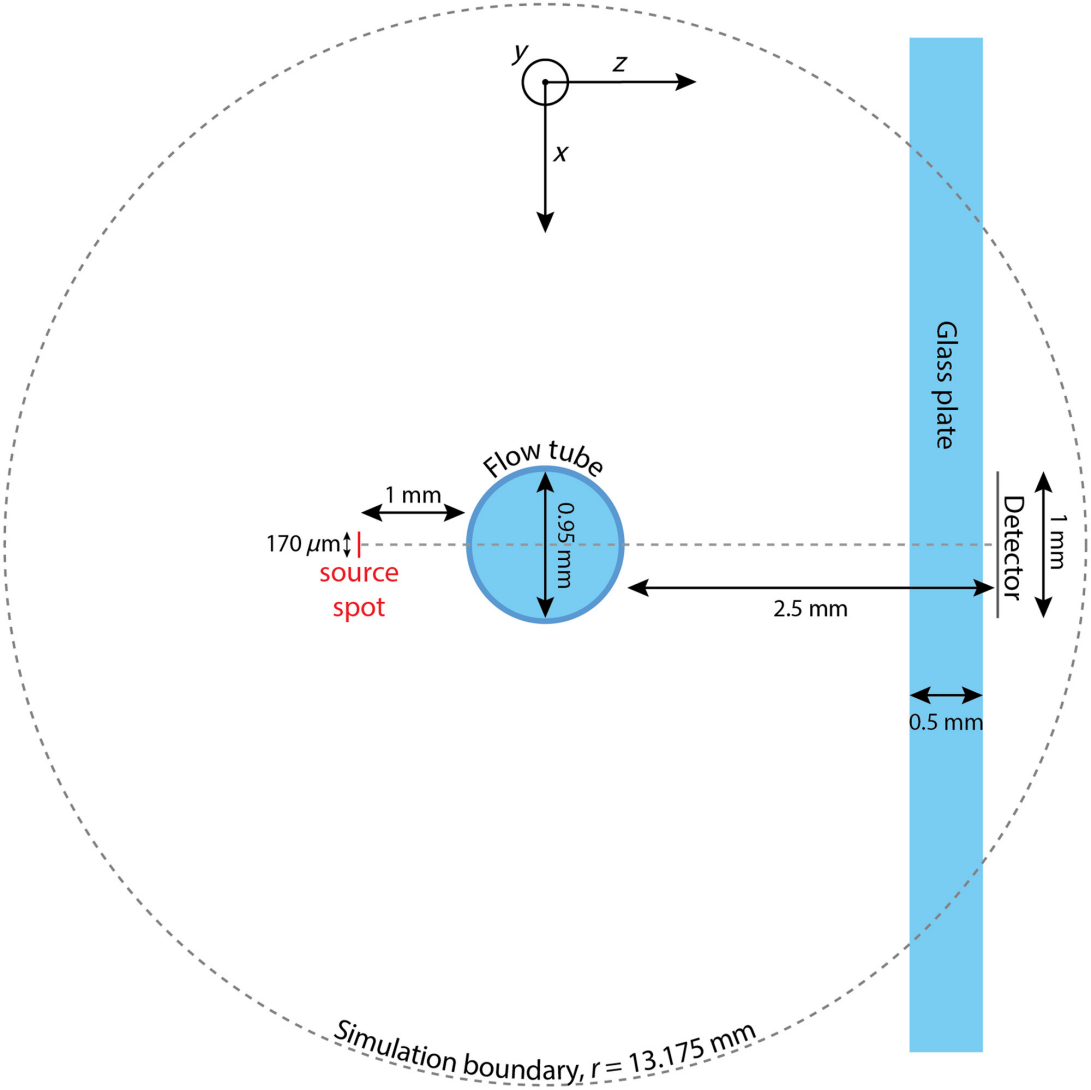
View of the simulation geometry in the x−z plane, showing the simulation boundary, source spot, flow tube, glass plate and detector.

At the start of the simulation run, the photons are given initial positions ($\vec{r}$) and propagation direction unit vectors ($\hat{k}$), which are dictated by the source structure. The photons also have a polarization unit vector ($\hat{p}$), for which the default state is parallel to the y–z plane (see Fig. 2), though it can also be user-defined. The polarization is always perpendicular to k^. The vectors $\overset{\to }{r}$, $\hat{k}$, and $\hat{p}$ for N photons are thus given in 3×N arrays, where each column of three then corresponds to the x, y, and z components of the relevant vector for a single photon. At the start of the simulation, each photon has a weight of 1. The weight decreases according to the Lambert–Beer law after each propagation step in media with non-zero absorption. The Doppler shift of each photon is initialized at 0 and is updated each time a photon scatters in a dynamic medium (see Sec. 2.1.6).

#### 2.1.3 Start of main simulation loop

Once the initial states of the photons have been defined, the main simulation loop begins, which will continue until all the photons have either been absorbed or have escaped the simulation region. At the start of the main loop, propagation distances for each photon are calculated from the inverse CDF [Eq. (3)], which is derived from the negative exponential PDF [Eq. (2)] of the photon path length based on the scattering coefficient of the current medium.

$$P(d)=e^{-\mu_{s}d},$$ (2)

$$i\mathrm{CDF}(\chi )=\frac{-\ln(1-\chi )}{\mu_{s}},$$ (3)

where $\mu_{s}$ and d are the scattering coefficient and path length, respectively, in meters, and χ is a uniformly distributed random number between 0 and 1. Once the path lengths are set, the positions of the photons can be updated.

#### 2.1.4 Photon propagation

There are three scenarios for the propagation of a photon in the program: 1) it can hit a material interface (see Sec. 2.1.5), 2) propagate the full path length and scatter (only in a medium with $\mu_{s}\neq 0$), or 3) hit the simulation boundary and escape. In all cases, the first step is to calculate the distances, for all photons, to all material interfaces that their path intersects. Derivations of the intersects between lines and arbitrarily oriented cylinders can be found in Ref. 33. If the distance to the nearest interface that is intersected is smaller than the photon’s path length, it is placed on that material interface at the intersect. If this is not the case, the photon is allowed to propagate its full path length and will be scattered at a later step of the program. Finally, if the photon neither intersects a material interface nor propagates a full path length before escaping the simulation boundary, it is placed on the boundary, stored in a structure for all escaped photons, and removed from the next iteration of the loop. Once the propagation step is completed, the weights of all photons are updated by use of the Lambert–Beer law for the travelled distance and the absorption coefficient of the medium they are currently in. If a photon’s weight drops below a user-definable value n (default 0.1), it has a 1−n chance of being absorbed, following the Russian roulette approach also used in MCML.^21^ If it is absorbed, its final state will be saved in the same manner as a photon that escapes the simulation, and it will be removed from the next iteration of the loop. If it is not absorbed, the weight is increased and the photon propagates again the next time around. In the default setting, the weight of a surviving photon is increased to 0.5, but the value can be user-defined. In setting up a simulation, the user may select a subset of the photons that they wish to save, as well as which photon properties are to be saved. In this manner, a detector can be defined, discarding any photons that do not hit it. Other selection criteria can also be applied, such as only saving photons scattered a certain number of times. By only saving the photon properties the user is interested in, usage of storage space and memory can be reduced. Should the user only be interested in photon propagation, but not in Doppler shift or polarization, those properties can be simply discarded for each detected photon.

#### 2.1.5 Crossing material interfaces

For all photons that have intersected with a material interface and are placed at the interface, the reflectivity (R) is calculated using the Fresnel equations. The reflectivity is based on $\hat{k}$, the unit vector $\hat{n}$ normal to the interface at the intersect, and the polarization state of the photon. From the reflectivity values, a uniformly distributed random number, χ, between 0 and 1 is drawn for each photon. If χ≤R, the photon is reflected, and $\hat{k}$ is updated accordingly. If χ>R, the photon crosses the interface, enters a new medium, and is refracted, updating $\hat{k}$ following Snell’s law in vector form.^34^ The path lengths are then recalculated for any refracted photons for the new medium that they crossed into, again using Eq. (3).

#### 2.1.6 Scattering and Doppler shift

A photon that propagates its full path length without leaving a medium and without being absorbed is scattered. First, a scattering angle θ and scattering azimuth ϕ are generated for each photon by interpolation in the inverse CDFs described in Sec. 2.1.1. Once $\hat{k}$ is updated, the orientation of $\hat{p}$ also has to change, which is done according to the derivation of Negus and Drain, Eqs. (7a) and (7b) in their paper. The scattered E-field amplitudes perpendicular and parallel to the scattering plane are calculated and used to calculate a resultant polarization vector. The polarization after scattering depends on the polarization of the incoming photon and the scattering direction, *via* the polarization-dependent phase function. Finally, for each photon located within a flowing medium, the local fluid velocity ($\vec{v}$) is calculated from the user-defined flow velocity distribution, and the Doppler shift is updated as

$$f_{D,i}=-\overset{\to }{v}\cdot \overset{\to }{q}+f_{D,i-1},$$ (4)

where i indicates the current iteration number, and

$$\vec{q}=\frac{n}{\lambda }({\hat{k}}_{i}-{\hat{k}}_{s}),$$ (5)

with n indicating the medium refractive index, λ indicating the wavelength of the light in meters, and ${\hat{k}}_{i}$ and ${\hat{k}}_{s}$ indicating the incoming and scattered direction unit vectors, respectively.

#### 2.1.7 Generating simulated Doppler power spectra

In traditional LDF, the autocorrelation of the spectrum of Doppler shifts is used to generate the power spectrum, as it would be seen on a photodetector.^8^^,^^29^^,^^36^ An important assumption that is usually made is that each point on the detector receives the same amount of light for each Doppler shift. Furthermore, any non-Doppler shifted light present on the detector is assumed to have undergone multiple scattering in the tissue matrix, homogeneously distributing the nonshifted light over the detector.^27^ These assumptions allow for the treatment of the detector as though it were a single point; the Doppler shifts of all photons can be considered together, and a single autocorrelation suffices. Although this generally holds for tissue in a backscattering geometry, with randomized flow directions due to the presence of a large number of microvessels in the detection volume, this is not the case for a single flow tube in transmission.

In the case of a clear glass tube with a scattering liquid flowing in a well-defined direction, several causes break the assumptions required for the above approach to work unmodified. Most importantly, because we are only considering a single tube with a well-defined flow direction, the assumption that each Doppler shift value is homogeneously distributed on the detector cannot hold. From Eq. (4), it becomes apparent that for low scattering, the positive Doppler shifts will be more present on the upstream portion of a detector centered on the incoming light spot, whereas negative shifts will be found more on the downstream portion. The power spectrum is built up from the difference frequencies between the various Doppler-shifted fields, which are expressed as speckles fluctuating in intensity over time on the detector. Only difference frequencies associated with the same speckle can actually be present in the detected signal and thus in the power spectrum. Simulation results shown in the Supplementary Material (Fig. S.1) indicate that the Doppler shift on the detector does indeed strongly depend on lateral position, along the fluid flow, whereas the elevational dependence is weak. In addition, nonshifted light is only present as nonscattered light on the detector. If the ballistic light exiting the tube forms a spot smaller than the detector diameter, the detection can only be said to be heterodyne inside that spot, whereas outside, the homodyne signal dominates. As long as the sample is low-scattering, the heterodyne signal dominates. As the sample becomes more scattering, the contribution of the ballistic spot becomes weaker until it vanishes.

In light of the above, we take the following approach for the generation of power spectra from the simulated Doppler-shifted photons:

1. We only consider photons falling on a user-defined detector surface.
2. We split up the detector into suitably sized strips perpendicular to the flow direction to account for the location dependence of the Doppler shift on the detector.
3. Per strip, we divide the Doppler spectrum into an appropriate number of bins.
4. We build the power spectrum bin by bin: calculate the interference signal for all photon pairs across bins in the Doppler spectrum separated by the difference frequency of the associated power spectrum frequency bin, also taking the effect of polarization into account.
5. Once completed for all strips, we sum all power spectra and divide by the number of strips.

To better compare the measured and simulated power spectra, the simulated spectra are normalized. Hereto, the simulated spectrum is first divided by the frequency-weighted sum of its values and then multiplied by the frequency-weighted sum of the corresponding measured spectrum. As a final step, white noise is added to the part of the simulated spectrum that falls below the noise baseline of the measured spectra. The simulated noise has an amplitude that is determined by the standard deviation of the noise in the measured spectra.

#### 2.1.8 Simulation geometry

Figure 2 shows an overview of the simulation geometry, which corresponds to the experimental setup described in the next section. A 170  μm diameter light source, with an NA of $2.5\cdot 10^{-3}$, is positioned in air 1 mm outside a soda lime glass tube with an outer diameter of 1.35 mm and an inner diameter of 0.95 mm. The medium inside the tube is water (n=1.33, λ=633  nm), with suspended polystyrene beads of either 1.0, 1.5, or 3.0  μm diameter and n=1.586 (λ=633  nm). From the bead sizes and the refractive indices of both beads and water, scattering phase functions are calculated based on Mie theory, using the MatScat package for MATLAB.^32^^,^^37^^,^^38^ For each bead size, low, moderate, and high $\mu_{s}$ values corresponding to those of actual samples (Table 1) are simulated. Laminar flow profiles corresponding to volume flow rates of 0.0, 0.1, 0.5, 1.0, 5.0, 10.0, and 15.0  mL/min are defined in the sample medium inside the tube. These values correspond to average flow velocities of 0, 0.0026, 0.013, 0.026, 0.13, 0.26, and 0.39  m/s, respectively. A random, Brownian component is also added to the flow profiles based on the particle size and room temperature conditions.^39^ In our case, the Brownian component has a typical magnitude of around 0.002  m/s and an isotropically distributed random direction. This means that here, for all but the lowest flow situation, the effect of Brownian motion is negligible. Early tests found only minimal differences between power spectra with and without Brownian motion, likely owing to the random directions of the Brownian component. In general, however, for smaller particles and/or lower flow rates, Brownian motion could indeed come into play. For completeness, we therefore kept the effect of Brownian motion in the simulations. Corresponding to the detector used in the experiment described in the next section, a 0.5 mm thick glass plate is placed at 1.8 mm from the tube outer surface. A 1 mm diameter circular detector is placed 0.7 mm behind the glass plate, centered on the light source. The simulation boundary is a cylinder with a radius of 13.175 mm and a length of 5 mm. Each simulation is run in the GPU mode until $5\cdot 10^{6}$ photons have hit the detector. For the highest scattering samples of the smallest particles, a simulation for a single flow rate takes ∼3  h on an NVIDIA T600 laptop GPU. For completeness, we have performed a comparison between MC-Doppler and MCmatlab^23^ on simulations of nondynamic light scattering. These results are presented in Sec. S.2 in the Supplementary Material and show excellent agreement between both simulation platforms.

**Table 1:** Overview of sample requirements, preparation outcomes, and measurement settings.

	Bead diameter								
	1.0 μm			1.5 μm			3.0 μm		
Target $\mu_{s}$ (${\mathrm{mm}}^{-1}$)	1.0	5.0	10.0	1.0	5.0	10.0	1.0	5.0	10.0
Target beads (% v/v)	0.026	0.130	0.260	0.028	0.140	0.280	0.116	0.590	1.160
Actual beads (% v/v)	0.028	0.140	0.290	0.032	0.160	0.311	0.116	0.560	1.166
Calculated $\mu_{s}$ (${\mathrm{mm}}^{-1}$)	1.07	5.35	11.08	1.16	5.82	11.30	0.99	4.86	10.01
Actual $\mu_{s}$ (${\mathrm{mm}}^{-1}$)	0.82	5.67	8.99	1.18	5.53	9.43	1.11	5.18	9.11
Measurement settings per sample									
Measurement gain	5×	25×	125×	5×	25×	25×	5×	25×	12×
Sampling rate (MHz)	1.0	2.5	2.5	1.0	1.0	2.5	2.5	2.5	2.5

### 2.2 Flow Measurements

#### 2.2.1 Setup

**Fig. 3:**
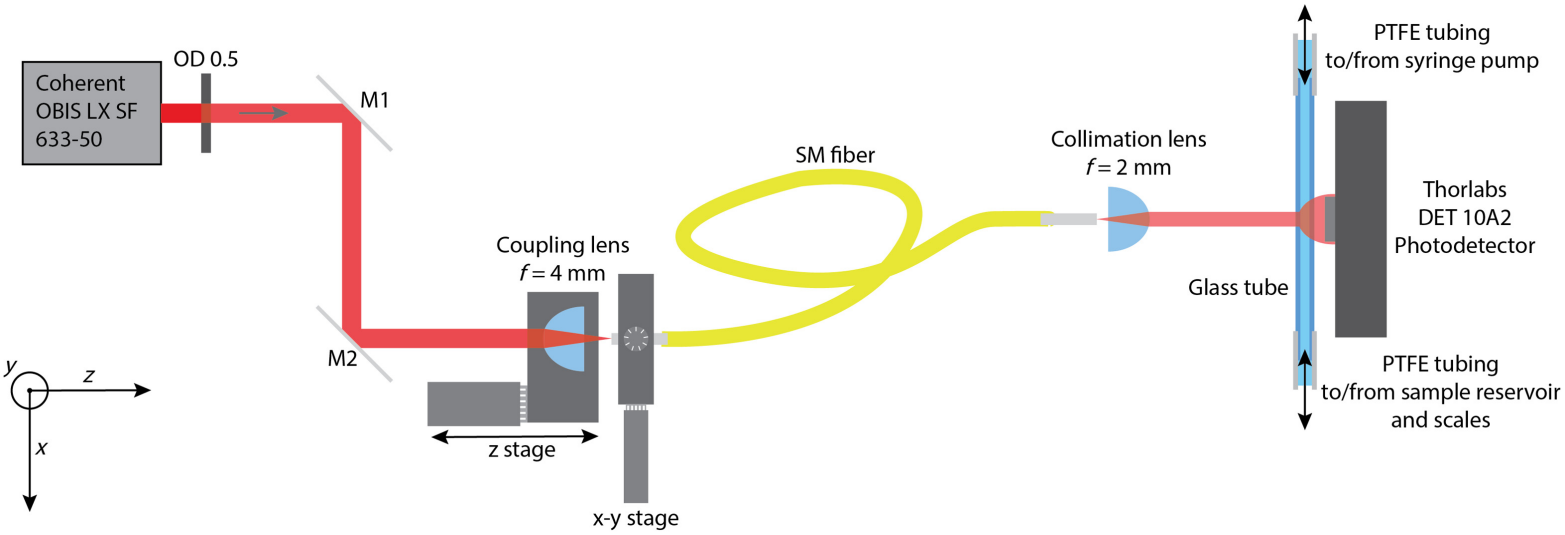
Schematic of the experimental setup, showing the laser, alignment mirrors, aspheric coupling lens on a z-stage, fiber input on an x−y stage, fiber output with adjustable collimation lens, sample flow tube, and photodetector.

Figure 3 shows a schematic of the experimental setup for through-transmission laser Doppler measurements. A 633 nm wavelength narrow-linewidth continuous-wave laser (OBIS LX SF 633-50, Coherent Inc., Saxonburg, Pennsylvania, United States) emits 50 mW of optical power. To prevent damage to the optical fiber, the output is limited with a neutral density filter of OD 0.5 (Thorlabs, Newton, New Jersey, United States). The beam is aligned on the coupling lens using a pair of tiltable silver mirrors (M1 and M2). The coupling lens is an aspheric singlet (C340TMD-B, Thorlabs, Newton, New Jersey, United States), with a 4 mm focal length, chosen to best match the NA of 0.10 to 0.14 of the optical fiber (P1-630Y-FC-1, Thorlabs, Newton, New Jersey, United States) for the 1 mm diameter (1/e) laser beam. The fiber output is directly connected to an adjustable fiber collimator (CFC2-B, Thorlabs, Newton, New Jersey, United States), which produces a collimated beam with a 170  μm 1/e diameter at the soda lime glass sample tube (Hirschmann Laborgeräte, Eberstadt, Germany). The fiber output with a collimating lens is placed in a cage system–mounted x–y translation stage (CXY1A, Thorlabs, Newton, New Jersey, United States). The sample tube is mounted in a 3D printed cage system–compatible mount, which is rigidly connected to the fiber output and collimating lens.

The sample tube has a 60 mm length, 0.95 mm inner diameter and 1.35 mm outer diameter, and n=1.52 (λ=633  nm). The liquid sample is transported to and from the glass tube via PTFE hoses (10014661, Eriks B.V., Rotterdam, the Netherlands), with an inner diameter of 1 mm and an outer diameter of 3 mm. By heating the hoses, inserting the glass tube, and allowing them to cool, an airtight seal is realized, ensuring reliable and leak-free pumping of the sample fluid. One of the PTFE tubes leads to a syringe pump (Aladdin 1000, World Precision Instruments, Sarasota, Florida, United States), which withdraws the sample from the sample reservoir on the other end of the tubing. The sample reservoir is placed on a digital scale (Ranger 3000, Ohaus, Parsippany, New Jersey, United States) to monitor the mass flow rate and compare it with the set volume flow rate of the syringe pump.

The detector (DET10A2, Thorlabs, Newton, New Jersey, United States) is placed on the far side of the glass tube, centered on the collimated beam. Depending on the signal amplitude, the photocurrent from the detector is amplified, 5×, 25×, or 125× by an RF amplifier (SR445A, Stanford Research Systems, Sunnyvale, California, United States), and then displayed on a digital oscilloscope (DPO 3014, Tektronix, Beaverton, Oregon, United States), from which the signals are saved for later processing.

#### 2.2.2 Sample overview

A total of nine suspensions of dry-form polystyrene beads (PolyBead Microspheres, dry form, 1.0, 1.5, and 3.0  μm diameter, Polysciences, Warrington, Pennsylvania, United States) with known optical properties are used. For each bead diameter, three suspensions in ultrapure Milli-Q water are prepared, aiming at scattering coefficients $\mu_{s}$ of 1, 5, and $10\;{\mathrm{mm}}^{-1}$. The required concentrations are calculated from Mie theory, using MatScat. Table 1 shows an overview of the calculated $\mu_{s}$ values based on the bead concentrations in volume percentage (%v/v).

Bead suspensions are sonicated in an ultrasonic cleaner (2510MT, Branson Ultrasonics, Brookfield, Connecticut, United States) in 5 to 15 cycles of 15 min, with 5 min breaks in between to prevent excessive heating of the samples. During the breaks, the samples are inverted a few times and placed on a vortex mixer (Vortex-Genie 2, IKA Scientific Industries, Wilmington, North Carolina, United States) for 20 s each, to further promote suspension of the beads. This process is repeated until no more bead clusters can be seen by eye, at which point bright field microscope images are taken for a more detailed assessment. The measured values of $\mu_{s}$ all samples, from a collimated transmission setup previously presented by Morsink et al.,^40^ are also shown in Table 1. The measured values are used in the simulations.

Once suspended and free from clusters, the samples are sealed and stored in a refrigerator at 7°C to minimize water evaporation. Prior to the experiment, samples are sonicated again for 10 s and warmed to room temperature.

#### 2.2.3 Measurement protocol and data processing

For each of the nine samples, a range of steady flow rates between 0 and 15  mL/min is applied using the syringe pump. Although more flow rates were measured, only a representative selection is shown in the results section. The flow rates for the syringe pump are calibrated using the digital scale to record the mass flow and calculating the volume flow rate from that.

Each sample is measured with the maximum gain that can be applied without overloading the RF amplifier; the gain settings and sampling rates used for each sample are also shown in Table 1. The temporal signals are taken with a sampling rate that will ensure inclusion of the highest frequency components of the signals as determined during test measurements on each sample at a high flow rate. The total number of samples in a time signal is kept equal, irrespective of the sampling rate, at $10^{5}$ samples.

To obtain power spectra from the signals, the time signal is cut into 128 segments of equal length, after which the FFT is taken for each segment. These 128 spectra are then averaged to alleviate noise due to the random nature of the speckle amplitude fluctuations that constitute the laser Doppler signal. Finally, the spectrum at zero flow is subtracted from this intermediate spectrum, yielding the Doppler power spectrum.

## 3 Results

### 3.1 Simulated Doppler Power Spectra

**Fig. 4:**
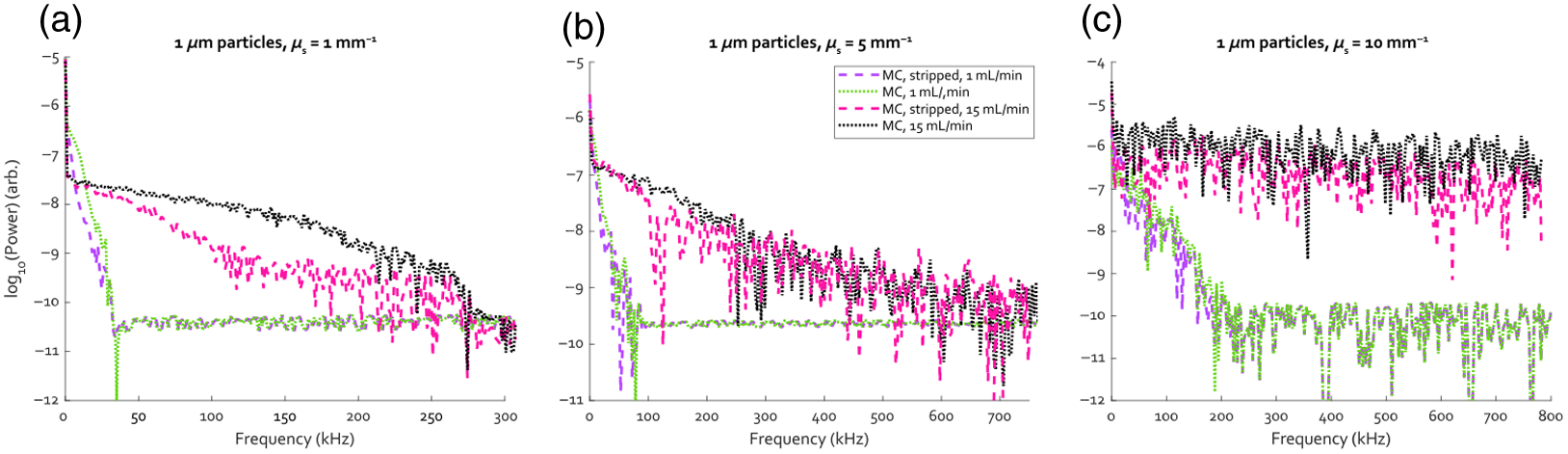
Comparison between simulated power spectra generated with the assumption of homogeneously distributed Doppler shifts (dotted) or spatially dependent Doppler shifts (dashed). (a) The spectra for flow rates of 1 and 15  mL/min for 1  μm particles with $\mu_{s}=1\;{\mathrm{mm}}^{-1}$, (b) with $\mu_{s}=5\;{\mathrm{mm}}^{-1}$ and (c) with $\mu_{s}=10\;{\mathrm{mm}}^{-1}$.

Figure 4 presents simulated power spectra of the 1  μm beads, at flow rates of 1 and 15  mL/min for scattering coefficients of $1\;{\mathrm{mm}}^{-1}$ in Fig. 4(a), $5\;{\mathrm{mm}}^{-1}$ in Fig. 4(b), and $10\;{\mathrm{mm}}^{-1}$ in Fig. 4(c). The dotted lines show the spectra when the detector is treated as one strip, on which the photons in each Doppler shift bin are distributed homogeneously, whereas the dashed lines show the result when the detector is divided into three vertical strips of equal width. Higher numbers of strips were also tested, but the spectra already converged at three, which keeps processing times to a minimum. For the lowest scattering sample, significant narrowing of the spectrum occurs when the spatial dependence of the Doppler shift is taken into account. As multiple scattering begins to dominate for more highly scattering samples, the difference between the two approaches starts to disappear, with minor differences still present in Fig. 4(b) and virtually none in Fig. 4(c). For consistency purposes, all subsequent simulated power spectra are processed by dividing the detector into three strips, regardless of the sample properties.

### 3.2 Comparison of Simulations to Experiments

**Fig. 5:**
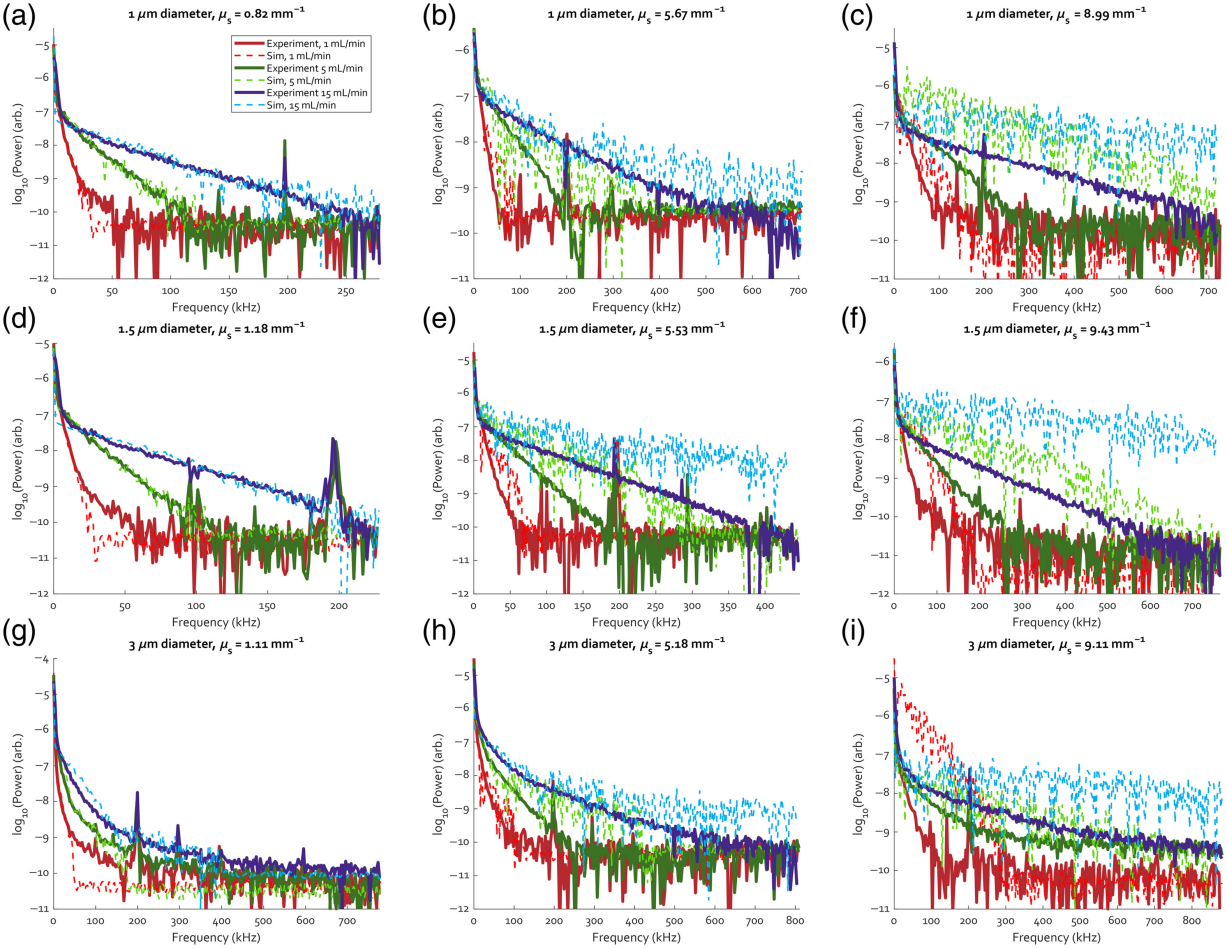
Measured and simulated Doppler power spectra for flow rates of 1, 5, and 15  mL/min for the nine polystyrene samples. (a)–(c) The three concentrations of 1  μm beads in ascending order, (d)–(f) the 1.5  μm beads, and (g)–(i) the 3  μm beads.

Figure 5 shows the power spectra of all nine samples for three flow rates each, i.e., 1, 5, and 15  mL/min, plotted on a logarithmic scale. The solid lines are the spectra from the experiment, and the dashed lines are the corresponding simulated spectra. Each row of subplots corresponds to one particle size, with $\mu_{s}$ in ascending order from left to right. Within each subplot, a broadening trend in both the measured and simulated power spectra for increasing flow rate can be observed. All experimental spectra show several spikes, the largest of which is at 200 kHz, which are likely due to mechanical vibrations in the setup.

In the single-scattering regime with $\mu_{s}=0.82$ to $1.18\;{\mathrm{mm}}^{-1}$ as seen in Figs. 5(a), 5(d), and 5(g), the simulated spectra overlap with the measured spectra. As multiple scattering begins to take hold for $\mu_{s}=5.18$ to $5.67\;{\mathrm{mm}}^{-1}$, in Figs. 5(b), 5(e), and 5(h), the simulated power spectra match less well for frequencies above approximately 100 kHz, whereas at lower frequencies the match is still good. For the highest scattering coefficients of 8.99 to $9.43\;{\mathrm{mm}}^{-1}$ [Figs. 5(c), 5(f), and 5(i)], however, the simulated and measured power spectra do not agree well, with the broadening in the simulated spectrum more pronounced than in the measured spectra.

### 3.3 Discussion

We have created an open-source Monte Carlo simulation platform in MATLAB, MC-Doppler, which is customizable by users to expand for a large number of applications for anyone familiar with MATLAB with an interest in scattering optics. We have demonstrated MC-Doppler’s abilities by comparing through-transmission laser Doppler experiments to simulations for a set of flowing polystyrene bead suspensions. For scattering coefficients up to around $5\;{\mathrm{mm}}^{-1}$, the simulations match the experiments well.

From these first results, we can see that for low to moderate scattering, Monte Carlo models could be used to help determine the flow velocity in a tube with a simple setup consisting of a single light source and one detector. Although the effect of particle concentration and flow velocity on the behaviour of the power spectra is pronounced, the effect of particle size is less strong (see also the Supplementary Material, Sec. S.3). This bodes well for flow measurements on samples with unknown and variable particle size distributions, such as human milk.

As multiple scattering comes to dominate, the simulated Doppler power spectra are much broader than those of the corresponding measurements. A similar overestimation of broadening for Monte Carlo–simulated power spectra relative to measurements with increasing multiple scattering was previously observed by De Mul^27^ in a backscattering geometry. In that work, the authors consider the effects of a limited detector NA, the possibility of an unanticipated local oscillator, and the effect of speckle size depending on incidence angle on the detector, though none of these appeared to be the cause. With our detector NA of 0.83, virtually all incident photons in the simulation fell within this NA. In the high-scattering samples, 99% of the detected light is scattered, precluding a strong local oscillator. The effect of incidence angle on speckle size, and thus mean intensity of the corresponding signal components does not compensate for the overestimation of the simulated spectral broadening on its own either. The glass plate in the simulations was added specifically to take any angle-dependent reflections into account but induced no significant effect on the power spectra. In the simulations, we assumed the particles to be homogeneously distributed in the flow field, meaning $\mu_{s}$ is constant throughout the sample and the entire flow profile is represented. Particle migration within the flow, leading to concentration gradients, may, however, play a role in the observed mismatches between simulation and experiment for higher concentrations.^41^^,^^42^ Furthermore, higher concentrations of particles may in turn also influence the flow profile. Although it is currently unclear if this plays a role in our experiments, future work could take this into account and update the simulations. Another possibility is that the particles form aggregates, which could influence the scattering behaviour of the suspensions to narrow the spectra, as is observed. Although care was taken to thoroughly sonicate the samples and measure as soon as possible thereafter, it cannot be guaranteed that no aggregates are present. Future work could incorporate a modification of the simulated scattering phase function based on a particle aggregation model to provide an indication if aggregates play a role.

In the simulated spectra for a 1  mL/min flow rate in the three lowest scattering samples [Figs. 5(a), 5(d), and 5(g)], a sudden drop to the noise baseline can be seen just below 50 kHz. These drops are an artifact of the data processing as their location on the frequency axis corresponds to the half-width of the Doppler shift spectra used to generate the power spectra. Above these frequency values in the power spectra, the zero-centered peak in the Doppler spectrum no longer contributes to the signal. In all other simulated spectra shown in Fig. 5, this point falls below the noise level and is not visible, either due to a higher flow rate or more scattering. This processing artifact is discussed in more detail in the Supplementary Material (Sec. S.4).

Although this work shows MC-Doppler to be suited to simulations of laser Doppler measurements in through-transmission in a cylindrical tube, detectors and sources can be placed in any location and at any angle. This means that aside from being used to evaluate experimental results as we have done here, it can also be a tool for the design of optical setups. Because the code can also serve as a ray tracer due to the accurate handling of reflection and refraction, minor changes to the code could include optical elements such as lenses, mirrors, and polarizers.

As the simulated power spectra are accurate for scattering mean free paths on the order of the channel diameter, a library of spectra can be generated for a range of flow rates and flow profiles for a given channel geometry and size. With such a library, it may be possible to develop a compact, low-cost alternative to current methods for flow characterization in lab-on-a-chip systems. A system based on a single laser, input fiber, and photodiode would be easier and more affordable to implement than the more complex laser Doppler profile sensors currently in use.^14^ Any flow profile that can be expressed as a function of position in MATLAB, within system memory limitations, can be simulated with our platform, opening the door to integration with computational fluid dynamics as well.

Future work should focus on further development of the software and the experiments it is meant to support. Although Sec. S.2 in the Supplementary Material shows excellent agreement of nondynamic light scattering simulations between MC-Doppler and MCmatlab, a comparison between MC-Doppler and another Doppler-enabled MC tool is currently missing. This is mainly caused by the difficulty of precisely replicating our simulations in the currently available tools. This should be addressed in the near future. More complicated situations such as particle migration and polydisperse samples may require the use of variance reduction techniques such as importance sampling,^43^ already implemented in an experimental branch of MC-Doppler. Furthermore, the issue of the mismatch between simulated and measured Doppler power spectra at $\mu_{s}>5\;{\mathrm{mm}}^{-1}$ should be addressed. In addition, measurements and simulations on more complex samples with multiple particle sizes and concentrations should show the applicability of this technique to the characterization of real-world scattering liquids, including human milk.

## 4 Conclusion

We have developed a non-voxel-based Monte Carlo simulation program for the simulation of cylindrical geometries, including fully customizable phase functions, optical properties, flow profiles, and the generation of Doppler power spectra based on these properties. Our results show good agreement between simulations and measurements on low to moderately scattering samples with known particle size distributions and concentrations. This paves the way for the development of more sophisticated models to determine the flow rates of samples with more complex properties.

## Supplementary Material

10.1117/1.JBO.30.8.087002.s01

## Data Availability

The Monte Carlo code described in this paper is available on GitHub and included in Ref. 31. The data are available upon reasonable request to the authors.
