# Supplementary material for: Monte Carlo simulation platform for laser Doppler flowmetry
Source: J Biomed Opt. 2025 Aug 26;30(8):087002. doi: 10.1117/1.JBO.30.8.087002 (PMC12379725; doi:10.1117/1.JBO.30.8.087002)
Supplement: Supplementary file 1 [file JBO_030_087002_SD001.pdf]

Supplementary materials

S.1 Location dependence of Doppler shift values

Figure S.1 shows six 2D histograms of Doppler shift (y-axis) vs. x or y position on the detector (x-axis), with the colour indicating the base 10 logarithm of the number of photons at each pixel. The plots are generated with the simulation results for the 3 µm particles, the top row, Fig. S1 (a)-(c) show the relationship of Doppler shift to y-coordinate for increasing particle concentration. The bottom row, Fig. S1 (d)-(f) shows the relationship between Doppler shift and x-coordinate. The flow is directed parallel to the y-axis, with the negative y-axis being upstream, the positive y-axis downstream. These plots show that the precise Doppler shift of a photon is related to where on the detector it lands, which is why the Doppler power spectra are generated by averaging the spectra of multiple lateral strips of the detector. The results are only shown for 1 particle size as they behave in the same way for the 1 and 1.5 µm particles.

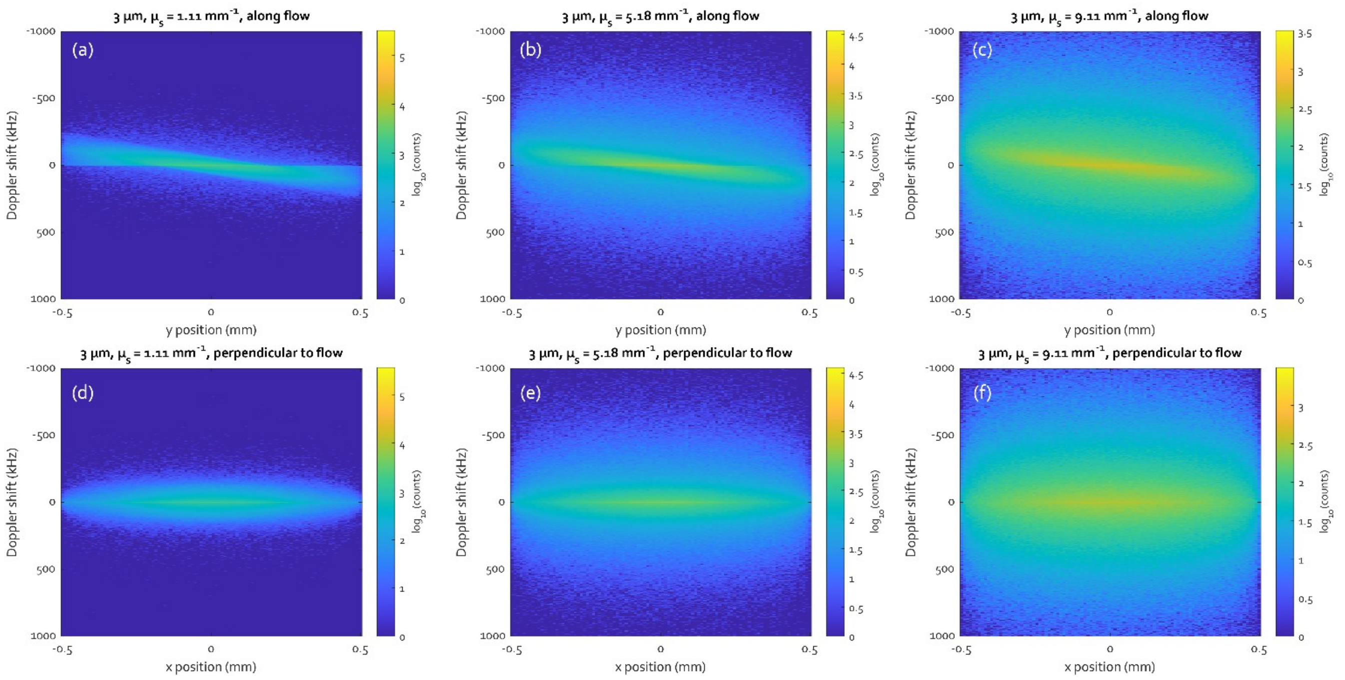

Figure S. 1 – Location dependence of Doppler shift on the detector. (a)-(c) show dependence of Doppler shift on the y-coordinate, (d)-(f) show the dependence on the x-coordinate. Flow is parallel to the y-axis.

S.2 Comparison of scattering behaviour between MC-Doppler and MCmatlab

Figure S.2 shows, on the left, our simulation geometry replicated on a 201x101x401 voxel grid in MCmatlab (ref. 23 in the main article). The illumination source is a pencil beam with a 633 nm wavelength in both cases, rather than the custom source used in the article. This is to ensure identical illumination in both cases. We simulated samples of 1.0 and 3.0 µm diameter polystyrene beads, with 10 concentrations from 0.02 to 1.0 % v/v. The phase function is identical in both cases, and calculated using MatScat as in the main article. The right-hand side of Fig. S.2 shows the values of μ_s based on through-transmission detection for both simulation platforms (asterisks for MCmatlab, squares for MC-Doppler), as well as the expected values from Mie theory as dashed lines. The simulations are found to be in close agreement. Deviations from Mie theory at higher particle volume fractions can be ascribed to the influence of multiple scattering.

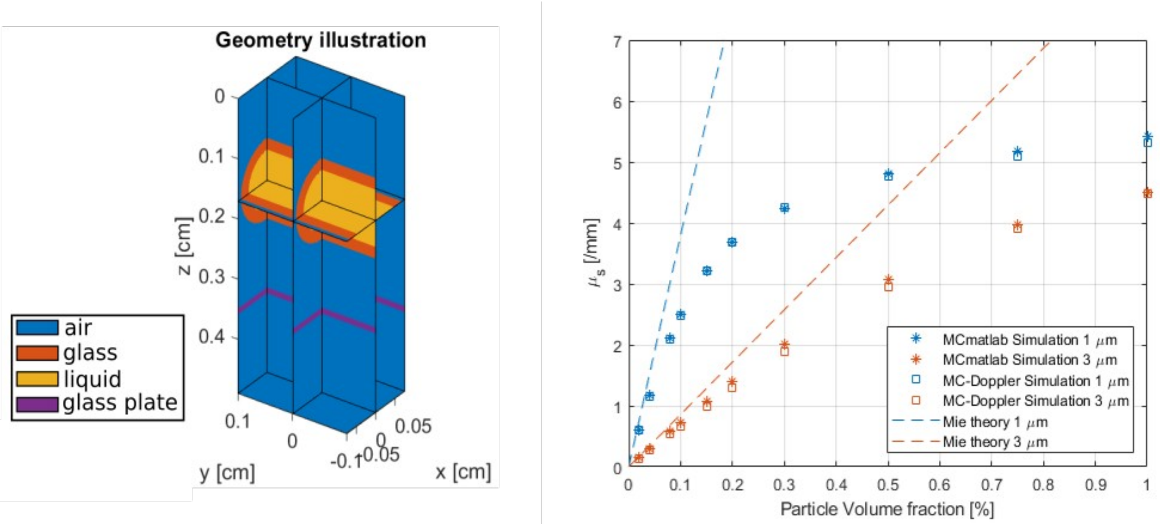

Figure S.2: Left: illustration of the simulation geometry in MCmatlab, replicating the situation in our MC-Doppler simulations as closely as possible, on a grid of 201x101x401 voxels. Right: comparison of the measured scattering coefficients in the forward direction for both simulations for two polystyrene samples at increasing concentrations. The dashed lines show the expected values from Mie theory, The asterisks the MCmatlab results and the squares the MC-Doppler results. Both simulations are in close agreement.

S.3 Dependence of power spectra on particle size

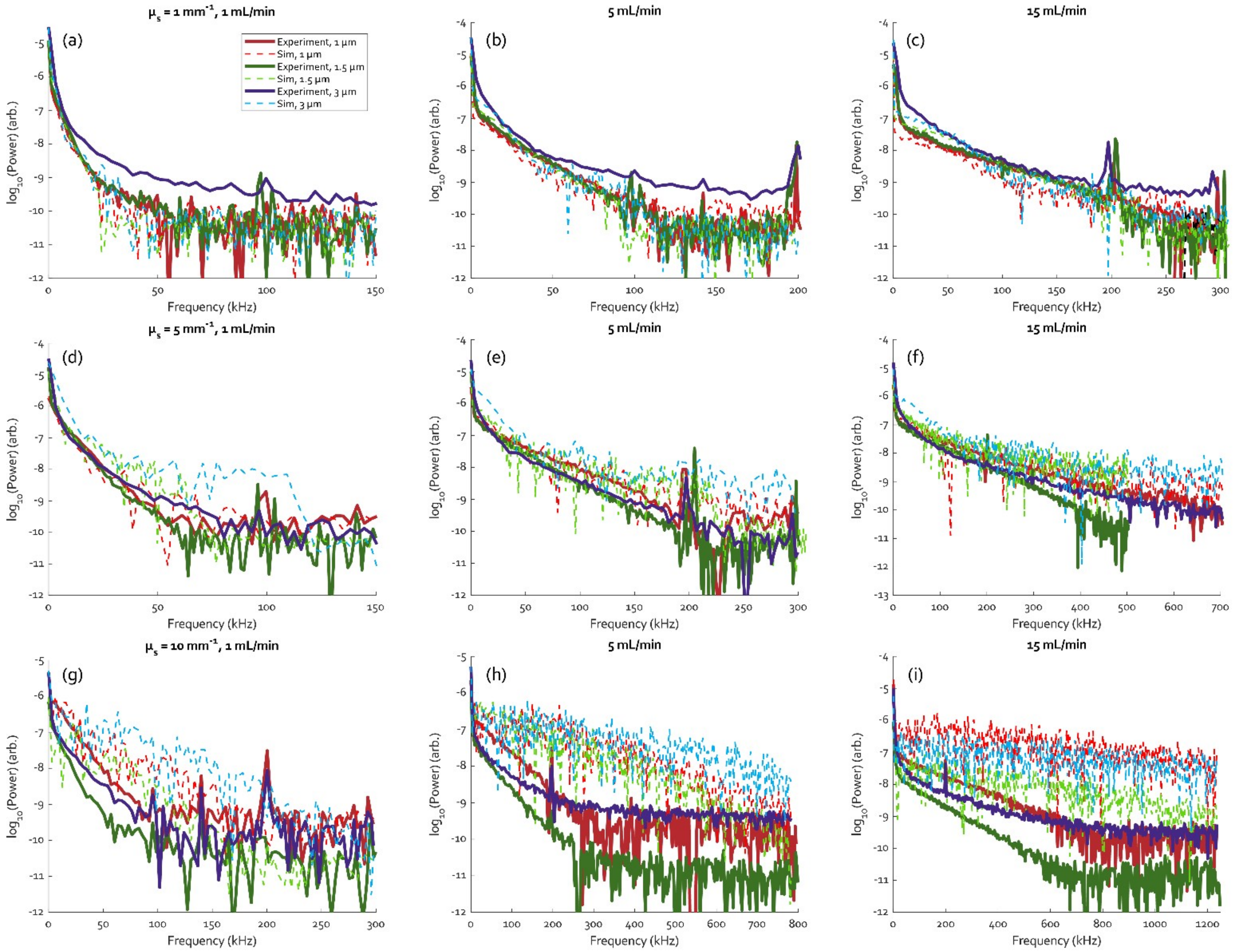

Figure S. 3 – Comparison of measured and simulated Doppler power spectra for a range of μ_s – values (ascending from top to bottom) and flow rates (ascending from left to right. (a)-(c) Show spectra for μ_s = 0.82, 1.18 and 1.11 mm^{-1}, for the 1, 1.5 and 3 µm particles respectively, at flow rates of 1, 5 and 15 mL/min. (d)-(f) show the same for μ_s = 5.67, 5.53 and 5.18 mm^{-1} for the 1, 1.5 and 3 µm particles and (g)-(i) show it for μ_s = 8.99, 9.43 and 9.11 mm^{-1} for the three particle sizes

Figure S.3 shows, per subplot, how the power spectra behave as a function of particle size for a given concentration and flow rate. While some differences can be observed, in particular between the 3 µm particles and the other two sizes for the experimental spectra, it seems there is not a strong dependence of the power spectrum properties on particle size. Concentration and flow rate have a stronger influence. To accurately determine an unknown flow rate from a measured power spectrum, it appears to be more important to have information on the scatterer concentration rather than the particle size distribution. Of course, to know more about a sample is always better, but Fig. S.3 at least indicates that the PSD is less for Doppler power spectra.

S.4 Processing artefact in simulated Doppler power spectra

Figure S.4 shows a side-by-side comparison of the experimental and simulated power spectra for the lowest concentration of then 1µm suspension at a flow rate of 1 mL/min, for two differently processed simulated spectra. Figure S.4 (a) shows the simulated spectrum processed under the condition that only bins of the Doppler shift spectrum containing 64 or more photons are used in calculating the power spectrum. Figure S.4 (b) shows the power spectrum calculated with no such restriction. In the former case a drop to the noise base line for the simulated spectrum can be clearly seen at 30 kHz, which is not present in the latter case. The drop is caused by a truncation of the Doppler shift spectrum at around 15 kHz, as this is the last bin containing 64 or more photons. This means that for any difference frequency larger than 30 kHz, the non-shifted photons no longer participate in the generation of those bins of the power spectrum. As those photons, especially in this low-scattering sample, constitute the majority, any bin in the power spectrum that they take part in generating will have a larger amplitude than others. By lifting the restriction on the minimum number of photons, the drop disappears. The source of the drop is well understood, and in most cases (higher particle concentration and/or flow rate) it falls below the simulated noise base line in any case. As the processing of the power spectra is substantially faster with the photon minimum imposed, we elected to process all simulated power spectra with the 64-photon minimum in place.

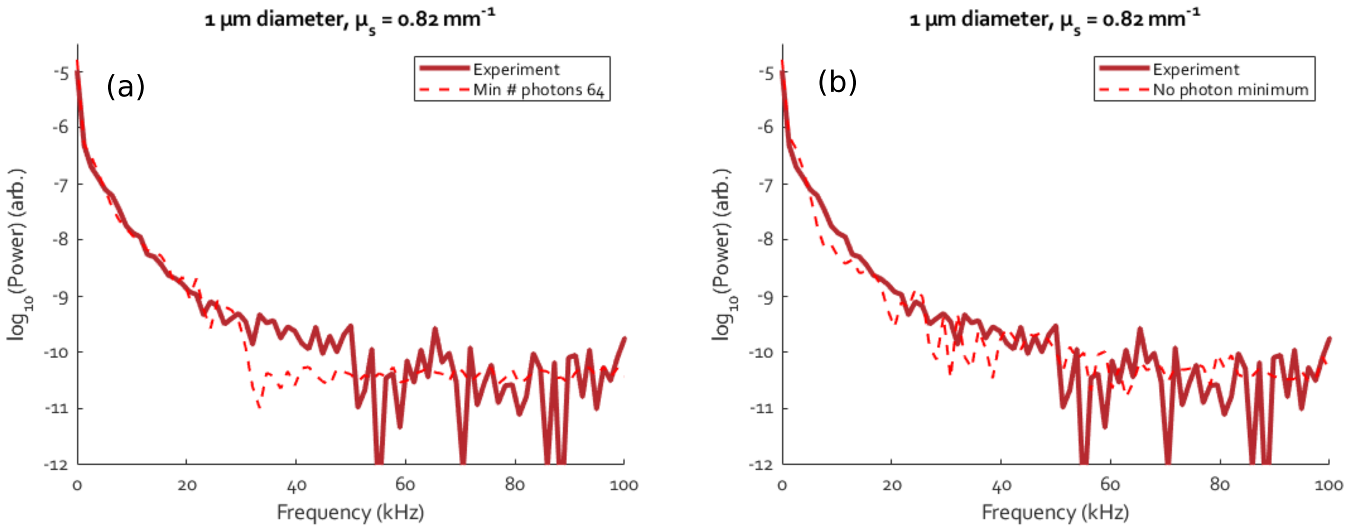

Figure S. 4 – Comparison of simulated power spectra for 1 µm particles with μ_s = 0.82 mm^{-1}, for a flow rate of 1 mL/min. (a) Shows the spectrum processed with a 64-photon minimum for the bins of the Doppler shift spectrum, while (b) shows the same spectrum without an imposed photon minimum. In both cases the experimental spectrum is also plotted, for comparison.
